# Citizen Science for public health

**DOI:** 10.1093/heapro/daw086

**Published:** 2016-12-08

**Authors:** Lea Den Broeder, Jeroen Devilee, Hans Van Oers, A Jantine Schuit, Annemarie Wagemakers

**Affiliations:** 1 National Institute for Public Health and the Environment, Bilthoven, the Netherlands; 2 Amsterdam University of Applied Sciences, Amsterdam, the Netherlands; 3 Tilburg University, Tilburg, the Netherlands; 4 VU University Amsterdam, Amsterdam, the Netherlands; 5 Wageningen University, Wageningen, the Netherlands

**Keywords:** public health, community participation, Citizen Science

## Abstract

Community engagement in public health policy is easier said than done. One reason is that public health policy is produced in a complex process resulting in policies that may appear not to link up to citizen perspectives. We therefore address the central question as to whether citizen engagement in knowledge production could enable inclusive health policy making. Building on non-health work fields, we describe different types of citizen engagement in scientific research, or ‘Citizen Science’. We describe the challenges that Citizen Science poses for public health, and how these could be addressed. Despite these challenges, we expect that Citizen Science or similar approaches such as participatory action research and ‘popular epidemiology’ may yield better knowledge, empowered communities, and improved community health. We provide a draft framework to enable evaluation of Citizen Science in practice, consisting of a descriptive typology of different kinds of Citizen Science and a causal framework that shows how Citizen Science in public health might benefit both the knowledge produced as well as the ‘Citizen Scientists’ as active participants.

## INTRODUCTION

In the Nieuw-West district of Amsterdam in the Netherlands a local community work organization proposes a project in which local residents, or ‘health ambassadors’, collect community views, requirements and concerns about health. One of the core values of this project is openness to all health topics that might come up. The project proponents discuss their ideas with the district council. The council is enthusiastic about the idea, but requests that the project should focus on overweight, as district obesity rates are high. It takes some time for the city council to abandon this preset idea: despite such high rates, obesity might not be the main priority of the residents in this community, and discussions with the community might yield a host of other important issues.

This anecdote illustrates the challenges posed by citizen participation in public health policy. On one hand, policy makers want to link up with community needs. Indeed, in the Netherlands, this wish underpins recent fundamental policy shifts, moving national-level responsibilities in the social and health domain to municipalities, and promoting participative approaches rather than professionally driven approaches.

On the other hand, policy development is a complex process. Policy makers refer to expert driven (epidemiological) data to underpin their priorities, as in the case described above, but the utilization of such knowledge is not a straightforward one-to-one implementation (Oliver *et al.*, 2014). Indeed, evidence is only one of many factors in the process of ‘juggling’ to create health promotion policies (de Leeuw *et al.*, 2014). As a result, policies may not appear to reflect citizens’ views and everyday experiences, even if they have been well considered, and citizens often do not recognize the policies as being relevant to themselves (Kooiker, 2011; Vaandrager *et al.*, 2011; Klaver *et al.*, 2014). This is probably even more so in those countries, such as the Netherlands, where local public health bodies are not governed by elected representatives of residents, but rather by appointed officials or civil servants. (For overviews of the different ways that countries organize their public health system, see for example the Health Systems and Policy Monitor of the European Observatory on Health Systems and Policies http://www.hspm.org/mainpage.aspx; 26 October 2016, last date accessed.) Citizen engagement in knowledge development, or ‘Citizen Science’, may prove useful. Citizen Science is defined as ‘the general public engagement in scientific research activities when citizens actively contribute to science either with their intellectual effort or surrounding knowledge or with their tools and resources’ (Socientize Consortium 2013). It first developed as a way to facilitate data collection, mainly in the natural sciences field. Since then, citizen science has developed in other work fields as well, for example in historical research, technology development, and the social sciences.

The aim of this paper is to explore the value of Citizen Science in public health. We begin by describing approaches in Citizen Science; discuss challenges for Citizen Science application in public health research; discuss how Citizen Science could promote better citizen engagement in public health policies and better health; and finally provide an evaluation framework.

## CITIZEN SCIENCE—A TYPOLOGY

To consider Citizen Science in public health we need to understand the different forms of this approach. An EU in-depth report (Science Communication Unit, 2012) describes three taxonomies classifying Citizen Science. Firstly, Roy *et al.* (Roy *et al.*, 2012) categorize Citizen Science by number and spread of participants (‘local’ or ‘mass’) and ‘thoroughness’ (investment of time and resources). Moreover, projects can be ‘contributory’ (led by experts), community-led or co-created. Or, in the terminology used by King *et al*. (King *et al.*, 2016), *for* the people, *with* the people, or *by* the people. (In literature about citizen science, activities are usually referred to as ‘projects’. We have noted that very often such activities are long-term and ongoing, and the term ‘process’ might be more suitable. However, in this paper we have adopted the usual terminology.)

Wiggins and Crownston (Wiggins and Crownston, 2011) classify projects according to aims: action, conservation, investigation, virtual or education. In ‘action’ projects, citizens and scientists jointly address local issues and concerns. ‘Conservation’ projects focus on managing natural resources. ‘Investigation’ projects focus on answering scientific questions. In ‘virtual’ projects, activities are carried out remotely. ‘Education’ projects aim at improving citizens’ knowledge.

The third taxonomy, by Haklay (Haklay, 2012), classifies Citizen Science projects by volunteer engagement levels. In ‘crowd sourcing’ projects (level 1), citizens are used as sensors or provide computing power. At level 2 (‘distributed intelligence’) citizens learn basic skills before they collect and interpret data. In ‘participatory science’ (level 3) citizens co-decide about research questions and types of data to be collected. Level 4 is ‘extreme’ Citizen Science’, or collaborative science. Although the term ‘extreme’, commonly used in the discourse about Citizen Science, seems to indicate a rare novelty, this is not necessarily so. For example, as early as in the late nineties, full engagement of all stakeholders, including citizens, in all research stages, was listed as one of the nine key principles of community-based participatory research (Israel *et al.*, 1998). For Citizen Science, ‘extreme’ indicates that citizens, are in full charge of the research and professionals are not included to any great extent (see, for example, Stevens *et al.*, 2014).

We have combined these three partly overlapping taxonomies into a single descriptive framework of Citizen Science project characteristics (Table 1). The first characteristic is the aim of citizen engagement. We have generalized the ‘conservation’ aim to a broader aim: the creation of ‘collective goods’. The second characteristic is level of participation, ranging from crowdsourcing to ‘extreme’ citizen science. The third characteristic is (geographical) size: either mass or local. From the original typologies we have excluded the on-site or remote (virtual) dichotomy. Most likely, in the near future the number of (partly) remote projects will grow, and it will be possible for local projects to be virtual as well. Moreover, this feature overlaps with ‘size’. We have also excluded the ‘thoroughness’ characteristic, as conditions and circumstances define research capacity need, which is not a core characteristic of citizen science projects in itself.

**Table 1:** Citizen Science descriptive characteristics

AIMS	Investigation: aimed at answering scientific questions Education: aimed at educational goals Collective goods: public health, management of infectious diseases, protect and manage natural resources. Action: citizens and scientists collaborate to address local concerns
APPROACHES	Extreme citizen science. Citizens in charge from problem definition, data collection and analysis, to interpretation and knowledge development Participatory science: Participation of citizens in problem definition and data collection Distributed intelligence Citizens as basic interpreters Volunteered thinking Crowd sourcing Citizens as sensors Volunteered computing
SIZE	Local Mass

Two examples—the ‘Galaxy Zoo’ project and the ‘Arctic Hunters’ project—show how this framework can be applied.

The ‘Galaxy Zoo’ project started in 2007 by asking citizens to help classify selected images of galaxies from the Sloan Digital Sky Survey, in order to increase research capacity. By 2009 over 200.000 people were involved. The project links up with other work fields, brought together in the ‘Zooniverse’, and educational activities were developed as a spin-off activity (Raddick *et al.*, 2010; Zooniverse, 2014a,b). On the basis of our checklist, we have characterized the project as 1Caii (aim: investigation, approach: distributed intelligence, citizen as basic interpreters, size: mass).

The Arctic Hunters project explores the potential of using digital resources to help Arctic coastal subsistence hunters to handle the impacts of climate change. This project combines traditional ecological (lay) knowledge with scientific expertise to develop a mobile technology embedding different ontologies and interpretations of sea ice. The technology is designed with the community and reflects their ways of hunting, their learning methods and their knowledge (ExCiteS, 2013; Mastracci *et al.*, 2013; Jennett *et al.*, 2014). On the basis of our checklist we have characterized the project with the code 4Aii (aim: action research, approach: extreme citizen science, size: local).

## REPORTED BENEFITS OF CITIZEN SCIENCE

Citizen Science is reported to yield benefits for scientists, policy makers, lay people and communities (Socientize Consortium, 2013). These can be grouped in three categories: increased research capacity, better knowledge and citizen benefits.

Increased research capacity, one of the main reasons for initial Citizen Science development (Rosner, 2013), refers to the need for larger quantities of data and the need for larger numbers of analyses. The main advantage, thus, is shared workload (Dickinson *et al.*, 2012; Socientize Consortium, 2013). Indeed, some authors consider labor-intensive projects requiring mass field data collection as being ‘ideally suited’ for citizen science application (Bonney *et al.*, 2009; Gommerman and Monroe, 2012; Socientize Consortium, 2013). An example, besides ‘Galaxy Zoo’, is a Dutch project where lay people help decipher 16th and 17th century letters provided to them through the project’s web system (Meertens Instituut, 2012).

A need for better knowledge, the second category of benefits, was another driver for Citizen Science development, building on the idea that adding lay, local and traditional knowledge to scientific knowledge could improve the scientific knowledge produced and therefore more effectively respond to complex societal problems (Irwin, 1995; Socientize Consortium, 2013). One reason is that this provides complementary data (Thornton and Maciejewski Scheer, 2012). In addition, the engagement of citizens may improve research strategies, or lead to novel research methods. Ottinger (Ottinger, 2010) describes how activist lay researchers of air quality showed that measuring peaks of emissions were as relevant for determining health risks as the usual procedure of monitoring long term averages. Thirdly, citizen engagement is viewed as producing more ‘socially robust’ knowledge (Nowotny *et al.*, 2001) that is acceptable and trustworthy to the general public, for example—in the field of knowledge development on cancer screening—acknowledging citizens’ feelings of doubt and fear regarding their decision whether to participate in screening programs.

The third category of benefits of Citizen Science is advantages for lay participants. A literature study regarding the benefits to citizens of participation in scientific research (Haywood, 2013) yielded a list of 10 main benefits. Case studies where a citizen science tool was applied in 10 neighborhoods in the USA, Latin America, and Israel (King *et al.*, 2016) showed similar benefits (Table 2). The first six citizen benefits in this overview are all related to the so-called ‘scientific literacy’: increased knowledge about the topic studied, insight into science in general and new skills and abilities—in short, ‘what citizens want to know’. Many citizen science projects include these as a project goal, and assess accomplishment (Cronje *et al.*, 2011). One example is the E-bird project which explicitly provides amateur bird watchers with new skills and knowledge—which in turn improves the quality of the data collected (Sullivan *et al.*, 2009).

**Table 2:** Claims about Citizen Science participant benefits (source: Haywood 2013; King *et al.*, 2016)

CITIZEN SCIENCE PARTICIPANT BENEFIT
Enhanced science knowledge and literacy (e.g. knowledge of science content, science applications, risks and benefits of science, and familiarity with scientific technology)
Enhanced understanding of the scientific process and method
Improved access to science information (e.g. one-on-one interaction with scientists, access to real-time information about local scientific variables)
Increases in scientific thinking (e.g. ability to formulate a problem bases on observation, develop hypotheses, design a study, and interpret findings)
Improved ability to interpret scientific information (e.g. critical thinking skills, understanding basic analytic measurements)
Science demystified (e.g. reducing the ‘intimidation factor’ of science, correcting perceptions of science as too complex or complicated, enhancing comfort and appreciation for science)
Strengthened connections between people, nature, and place (e.g. place attachment and concern, establishment of community monitoring networks or advocacy groups
Empowering participants and increasing self-efficacy (e.g. belief in one’s ability to tackle scientific problems and questions, reach valid conclusions, and devise appropriate solutions)
Increases in community-building, social capital, social learning and trust (e.g. science as a tool to enhance networks, strengthen mutual learning, and increase social capital among diverse groups)
Changes in attitudes, norms and values (e.g. about the environment, about science, about institutions)
Citizen scientists take action to influence policy and/or improve living environment
Citizen scientists gain access to broader (policy making) networks

Haywood and King both mention additional benefits that are less ‘cognitive’. They include community development, empowerment, and change of attitudes, values and norms, action to improve the environment, and engagement in policy making. It is reported that lay researchers start using and applying the knowledge and abilities acquired, and strive to change their environment or their behavior (Brossard *et al.*, 2005; Bonney *et al.*, 2009, 2014; Dickinson *et al.*, 2012; Socientize Consortium, 2013). Reportedly, the educational value of Citizen Science has helped reduce social exclusion (Socientize Consortium, 2013).

## FORERUNNERS OF PUBLIC HEALTH CITIZEN SCIENCE

Recently, the use of citizen science has been booming as a result of the need for mass data, growing confidence in and valuation of the input of lay people, and technological development (Goodchild, 2007; Raddick *et al.*, 2010; Roy *et al.*, 2012; Socientize Consortium, 2013). Apparently, Citizen Science is rare in public health: a recent overview of ‘good examples’ produced for the European Commission contained no public health-related Citizen Science projects (Socientize Consortium, 2013). It seems that the largest part of Citizen Science work is carried out in the fields of biology, conservation and ecology, although Citizen Science in other work fields may remain unpublished as it is not primarily focused on scientific gain (Kullenberg and Kasperowski, 2016). Indeed, some approaches in public health research strongly resemble Citizen Science. One of these is (participatory) action research, defined as a ‘participatory process concerned with developing practical knowledge, in the pursuit of worthwhile human purposes. It seeks to bring together action and reflection, theory and practice, in participation with others, in the pursuit of practical solutions to issues of pressing concern to people, and more generally the flourishing of individual persons and their communities’ (Reason and Bradbury, 2008). Participatory action research can definitely be seen as a citizen science approach. However, the two are not exactly the same: as the action part refers to the focus on taking action to bring about social change, addressing specific problems and developing interventions—or preparing decisions—to solve them (Minkler, 2000; Wagemakers, 2010), citizen science may also be carried out without such a preset focus on action. Furthermore, in the health sciences participatory research ‘is conducted by a coalition of researchers, community members, patients, health professionals or other stakeholders’ (Hughes, 2008, p. 385), resembling a strong involvement of citizens, similar to code **4A**(aim: action research, approach: extreme citizen science) (Table 1). In citizen science, citizens can also be engaged in research activities in other, less intensive, ways.

Another similar approach is ‘popular epidemiology’, in which laypersons join experts to collect—mostly environmental—data that lead to specific health outcomes (Brown, 2013), or ‘street science’, a process in which communities actively engage in problem definition, framing of research questions, and decision-making about study design (Corburn, 2005) Like (participatory) action research, these are, again, closely connected to social mobilization and problem solving. A related process is Health Impact Assessment (HIA), community engagement can be part of all steps in HIA (Mindell *et al.*, 2004; Hebert *et al.*, 2012; Chadderton *et al.*, 2013).

## CHALLENGES FOR THE APPLICATION OF CITIZEN SCIENCE IN PUBLIC HEALTH

The development of public health Citizen Science may build on the forerunners described above, and learn from them, in particular since public health issues are linked to our personal lives, and ethical considerations in research such as data ownership or informed (community or individual) consent are urgent in this field (Khanlou and Peter, 2005). Moreover, public health issues can be the topic of public dispute, for example, in the case of large-scale livestock farming, where the economic development of a region has to be weighed against possible health impacts in terms of environmental damage or zoonosis’ transmission risk. In such situations, the distinction between knowledge development and advocacy or political activism may become blurred. A sense of distrust in science as something that can be manipulated based on stakeholder’s preferences may then be the result, rather than a genuine dialogue and better understanding of science. Seeking connections between citizens and experts on the one hand, and safeguarding research quality on the other hand therefore requires carefully balanced management of Citizen Science research processes (Van Buuren *et al.*, 2007).

Apart from these fundamental issues, the application of Citizen Science in public health poses a number of additional, partly related, challenges when put to practice.

First of all: why would lay people be bothered to engage in scientific research? Studies of Citizen Science participant motivations show that people have different—sometimes multiple—reasons for participating (Raddick *et al.*, 2010; Roy *et al.*, 2012; Socientize Consortium, 2013). These include intrinsic interest in a topic, being part of a community, contributing/helping, learning, or the enjoyment of research activities. Citizen Science projects correspondingly use various engagement strategies (Greenwood, 2007, Dickinson *et al.*, 2012; Roy *et al.*, 2012; Socientize Consortium, 2013; Pocock *et al.*, 2014).

Such motivations resemble those found in a Dutch study on volunteers in health promotion (Fienig *et al.*, 2011): the wish to contribute to a greater (health) goal, personal development, the wish to help others when asked, and the wish to be an example and inspiration for others. Citizen science engagement strategies from other work fields may therefore work well in public health research.

Secondly: when lay people are engaged, do they really represent the group that needs to be represented, geographically, or socially? Brown, for example, observes that women often play an important role in popular epidemiology, which he ascribes to the (family) roles of women combined with a relationship-centered world view, and thus stronger awareness of the potential health impacts of toxic factors (Brown, 1997). Such over—or underrepresentation may impact on study results. In researcher-controlled Citizen Science projects gathering mass data, this need not be a major issue. Indeed, large scale research where citizens act as ‘sensors’ is already applied in epidemiology, for example, in studies in which people wear measurement devices (Wendel-Vos *et al.*, 2003). In small-scale and more participative studies, lay researcher selection may cause bias. However, it may also be an asset: lay researchers can access ‘hard to reach’ study populations as ‘peer researchers’ (Kamann and De Wit, 2012).

Thirdly: how to weigh the scientific and social value of citizen-generated knowledge? And: do volunteers have adequate capabilities and competences? Views of professionals and lay people—a systems view versus experienced reality—may be difficult to reconcile (Klaver *et al.*, 2014), and researchers sometimes disqualify lay research outputs as unscientific (Cohn, 2008; Ottinger, 2010). One solution may be training: often a part, and sometimes an aim, of Citizen Science projects (Cohn, 2008). Another solution would lie in enabling dialogue between scientists and lay people instead of ‘professionalizing’ lay people, widening research scope and generating information on community features that are key in understanding the community’s health problems (Brown, 2013). Such dialogue could even induce methodological innovation. For example, local residents who participated as lay researchers in Amsterdam, the Netherlands, stated that the number of children eating snacks outdoors reflect neighborhood health (den Broeder *et al.*, 2015); and that therefore, existing health indicators should be amended by new, observational, ones.

Fourthly, will citizen science *per se* promote participatory policy development? Projects with citizens as ‘sensors’, led by scientists with little connection to local issues, will not necessarily do so. However, more community-driven research may effectively empower people to participate in local policy making (Brown, 1997, 2013; Socientize Consortium, 2013). Citizens regard access to information and knowledge as a key condition for participation (Litva *et al.*, 2002) and a review about community-based health research in the USA showed that the more the community was in control of the research, the more community members took action to create better health (Cook, 2008). As participation in research activities may enhance a sense of community and develop new community values and norms (Haywood, 2013), this may also be true for public health research and therefore boost active health policy engagement. King *et al*, mentioned before, provided an example of the latter: the ‘Our Voice’ framework, a citizen science approach developed to assess healthy neighborhoods with residents. Within ‘Our Voice’ a digital tool was developed enabling citizen scientists, in particular people in underprivileged districts, to gather data, mostly on how their environment enhances or hinders physical exercise. King *et al* report that, in several cases where the tool was applied, citizen scientists afterwards undertook concrete actions to improve their environment and/or developed engagement in local policy making, including engagement at the request of local government (King *et al.*, 2016). Finally: can this approach really improve the health of the population? We think this is certainly possible. Citizen Science may not only increase participants’ ‘health literacy’, i.e. ‘the skills and capacities that enable people to exert greater control over their health’ (Nutbeam, 2008), and an important condition for adequate health behavior (van der Heide *et al.*, 2013). It may also enhance their ‘sense of coherence’, i.e. the degree to which they experience the world as comprehensible, meaningful, and manageable. A high SOC is reported to promote better health (Lindstrom and Eriksson, 2006). On a community level Citizen Science may promote community values and social cohesion; these are important factors that contribute to community health (Whitehead and Dahlgren, 1991).

**Fig. 1:**
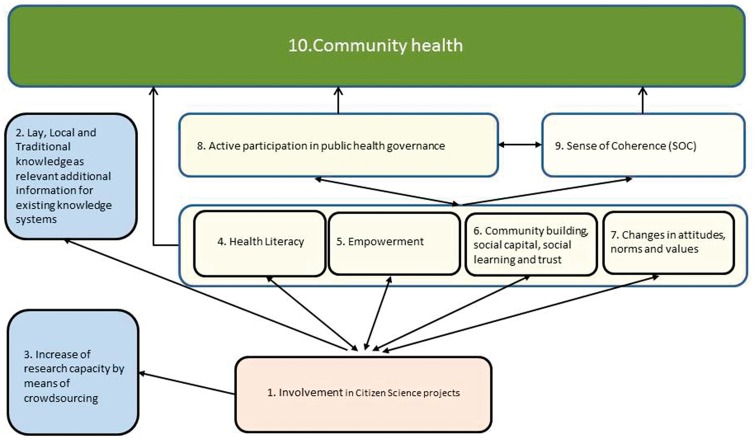
Effects of Citizen Science on health, health governance and knowledge system.

Figure 1 shows an overview of potential citizen science benefits, including both ‘better knowledge’ and advantages for citizens themselves and their health. HIAs, mentioned before, may illustrate how some of these benefits, such as resident empowerment, are actively pursued (Table 3). In relation to HIA, there is discussion about advantages and disadvantages of citizen engagement, resembling similar issues in Citizen Science in general; in particular issues regarding selection of participating citizens, their competences, and the value of knowledge gathered by or with citizens (Wright *et al.*, 2005). These issues and their potential scientific, political and ethical consequences must be addressed in Citizen Science practice, as they should be in HIA practice.

**Table 3:** Case examples of CS benefits in Health Impact Assessment

Bubble in Figure 1^a^	Case example
1. Involvement of citizens (residents)	Community representatives (Aboriginal community) participated in HIA Steering Group and decided on scope and methods of an HIA on a broad set of government measures to protect children and families (NTER). Health impact indicators are based on Aboriginal concept of health (*Australian Indigenous Doctors’ Association and Centre for Health Equity Training, 2010*).
2. Inclusion of lay and local knowledge	Community experiential knowledge was key to specifying relations between those social determinants considered meaningful by the community, individual and community mental health. The pathways thus developed served as a basis for an HIA on policy regarding the use of arrest records in employment decisions. (Todman et al., 2013).
3. Increased research capacity	Community representatives collect data about resident qualifications of current situation and experiences with earlier cut-downs on bus services (survey, interview) in a HIA on public transport (Alameda County Public Health Department, 2013).
4. Health literacy	Residents engaged in an HIA on local health hazard control policy were provided information about legal frameworks, policies and health hazards. They reported increased knowledge on health hazards, the social determinants of health, and the need to address these*(*Inmuong et al. *2011*).
5. Empowerment	Residents representing a local community assessed potential health impacts of a plan to create an outdoor recreation area nearby, using the local community health vision as a starting point. They prepared a set of recommendations providing points of attention and proposals to adapt the project plan*(*Eaton and Cameron, 2008).
6. Community building, social capital, social learning, trust	An HIA on a regional transport policy explicitly aimed at building co-working relations between community and different agencies. Evaluators of the HIA observed that some, though not all, community members thought this was accomplished *(*Kearns and Pursell, 2007).
7. Changes in attitudes, norms, values	HIA of remediation of a former industrial site included HIA training of community members. This resulted in a more positive attitude towards HIA*(*Lester et al. 2003).
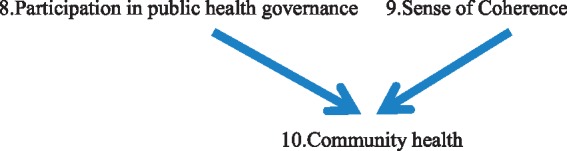	

a The numbers refer to the bubbles in Figure 1.

## FUTURE DIRECTIONS FOR PUBLIC HEALTH CITIZEN SCIENCE

Over the past years, various deliberative methods such as citizens’ juries and citizens’ dialogues have been developed to enable meaningful contributions by citizens to policy development (Maxwell *et al.*, 2003; Street *et al.*, 2014). However, although these methods facilitate the transfer of knowledge from experts to lay people, they are applied within short time frames, and therefore allow little space for joint knowledge development (Abelson *et al.*, 2003). Citizen Science engages people for a longer period of time, which may help to strengthen and sustain an active role for citizens, both in research and in the application of the knowledge produced in policy development.

Of course we need to be aware of the challenges and potential downsides of Citizen Science in Public Health. For example, how are local needs weighed against the needs of wider population groups, both geographically and socially? How to prevent tokenism, where participation of residents is used to provide a false impression of ‘democratic’ decision making? The approach still needs a lot of practice testing (Cronje *et al.*, 2011). Our framework, combining descriptive characteristics of Citizen Science (Table 1) and its potential effects on health, health governance and the knowledge system (Figure 1), presents a basis for studying, comparing and exploring the opportunities and limitations of public health Citizen Science. Such practice testing may yield practical guidance for public health Citizen Science, for example how to link up with local contexts, how to determine the appropriate level of citizen engagement, or how to ensure stakeholder commitment.

We believe that, despite all the questions and doubts, Citizen Science has much to offer for public health research. Citizen science as a way to collect data with lay people’s help may be particularly useful in the field of infectious diseases. For example, bird flu outbreaks may be more rapidly detected with the help of a network of citizen scientists such as hobby farmers or bird watchers. Lay people’s input can also be helpful for environmental health monitoring. A recent example is the I-Spex project in which thousands of citizen scientists submitted air quality measurements (Snik *et al.*, 2014), a mass crowd sourcing approach for a collective aim - in our table: 3Dii.

Citizen Science in public health can also inform local policy makers about residents’ perceptions and views, and provide access to lay knowledge. This may enable policy makers to address resident concerns, and ‘empower’ them to strike a balance between such concerns and other (health and other) priorities. But most importantly, Citizen Science, applied as an inclusive approach, has the potential to boost the participation of citizens in public health policy processes by increasing health literacy, empowerment and community cohesion, creating new attitudes and values, and producing a stronger sense of coherence.

## FUNDING

This work was funded by the Strategic Programme RIVM (SPR) of the National Institute for Public Health and the Environment in the Netherlands, grant number S/015026/01/CS.
